# Digestive Fermentation, Antioxidant Status, and Haemato-Biochemical Indices of Growing Rabbits Fed on Diets Supplemented with *Ziziphus spina-christi* Leaf

**DOI:** 10.1155/2020/9046862

**Published:** 2020-03-31

**Authors:** Suha Hashim Abduljawad

**Affiliations:** Department of Nutrition and Food Science, Faculty of Family Science, Taibah University, Al-Madinah Al-Munawarah, Saudi Arabia

## Abstract

The objective of the study was to assess the effect of diets supplemented with *Ziziphus spina-christi* leaf on digestive fermentation, antioxidant status, and haemato-biochemical indices of growing rabbits. *Ziziphus* leaves (ZLs) of *Ziziphus spina-christi* were collected from Sidr trees scattered throughout the city of Medina, Saudi Arabia. Three formulated diets containing 0, 10, and 20 g *Ziziphus spina-christi*/Kg diet as supplementation were offered *ad libitum*. The organic matter content of ZL was higher. Chemical compositions were comparable in all of the contents of the tested diet. Quantities of gas released from the control diet were higher, and then the gases released decreased significantly (*P* < 0.05) with the addition of ZL. The values of NH_3_-N were taken as the same trend. The addition of a high level of ZL to rabbit diets led to a decrease (*P* < 0.05) in the total count of bacteria as well as the number of *E. coli* and *Clostridium* spp. However, the number of *Enterococcus* bacteria was not affected by supplementation. Haemoglobin parameters of the control group and groups 2 and 3 were compared: white blood cell count and red blood cell count. These observations of total protein and albumin within the range of reference values were reported in healthy rabbits, while glucose significantly decreased with the addition of ZL and AST in the blood increased significantly. The values of TP, albumin, and ALT measurements showed no significant differences among groups fed on test diets. Significant differnces in serum immunoglobulins were observed between the groups, while the high levels of ZL supplement led to a significant (*P* < 0.05) increase in the serum IgA, IgG, and IgM levels. Antioxidants expressed as T-AOC, GSH-Px, T-SOD, and CAT in the blood of animals fed on diets containing high levels of ZL were significantly higher. Higher serum T-AOC, T-SOD, and CAT activities were observed in rabbits supplemented with a high level of ZL compared with the control group (*P* < 0.05). The supplementation of ZL tended to increase serum GSH-Px activity. The addition of ZL to rabbit diets led to an increase in dry matter intake. On the other hand, there was no significant change in the apparent digestion coefficient of DM, OM, CP, and fat. *Conclusion*. Dried ZL supplementation up to 20 g/Kg diet might improve the bacterial community, antioxidants, biochemical parameters and blood constituents of rabbits, and digestibility.

## 1. Introduction

Medicinal plants are used in reducing predisposition and managing diseases related to oxidative stress, such as cancer and hypertension, as they contain strong antioxidants such as flavonoids, anthocyanins, and alkaloids [1]. Using antibiotics or synthetic growth promoters in the regime diet of rabbits has become expensive. On the other hand, using natural substances in herbal plants could lead to the same function, without side effects and at less cost [2]. Furthermore, the efficiency of natural substances is comparable to industrial materials [3]. Disorders of gastrointestinal can cause death in 30–50% of the rabbit stock, which can also impact performances of rabbits [4].

Considering the continuous increase in the price of these materials, the need for improving the feeding process, the benefits of reduction of disorders caused by agitation of the gastrointestinal tract, and then the usage of these natural materials would be very useful. For resolving these problems, it is necessary to apply the growth promoters of natural extraction, which are capable of providing comparable efficacy and will not conduce to the cumulative contamination of the environment [5].

*Ziziphus spina-christi* is a subtropical plant known in the Kingdom of Saudi Arabia as “Nabq” or “Sidr” which is used for various medicinal purposes. The *Ziziphus* leaves (ZLs) contain various alkaloids, including ziziphine, jubanine, and amphibine, alpha terpinol, linalool, and diverse saponins. *Ziziphus spina-christi L* has been shown to have an activity against bacteria and fungi [6].

In this study, we set out to test the composition and functioning of the ecosystem of the digestive tract of rabbits when incorporating *Ziziphus spina-christi* in their diet. The objective of the study was to assess the effect of diets supplemented with *Ziziphus spina-christi* leaf on digestive fermentation, antioxidant status, and haemato-biochemical indices of growing rabbits.

## 2. Materials and Methods

The experimental work was carried out at the animal house of King Khalid Hospital, Kingdom of Saudi Arabia (KSA). *Ziziphus* leaves (ZLs) of *Ziziphus spina-christi* were collected from Sidr trees in the city of Medina, Saudi Arabia. After verification and authentication, a voucher specimen was deposited in the herbarium of the Department of Laboratory Sciences, College of Sciences Medina, Taibah University. Collected leaves were washed thoroughly with tap water, rinsed again in distilled water, and then dried in shade for up to twenty days. The dried leaves were crushed to a fine powder using a blender and kept until used.

### 2.1. Experimental Animals, Housing, and Diets

Three formulated diets containing 0, 10, and 20 g·ZL/Kg diet as supplementation were offered *ad libitum*, and residues were estimated to calculate actual feed consumption. The control group was fed on a diet without ZLs (ZL0); the experimental groups were fed on the same diet with 10 and 20 g·ZL/kg (ZL10 and ZL20, respectively). The chemical composition of the experimentally and differently tested diet is shown in Table 1. The experimental period lasted for 8 weeks (from 6 to 14 weeks of age, weighed 748.88 ± 8.04 gm). Thirty weaned male rabbits (New Zealand White) at the age of 6–7 weeks were allocated randomly into three groups of the same weight and age. During the experiment, the health status of the rabbits was checked daily in terms of vitality, digestive disorders, and mortality. Rabbits were raised in a well-ventilated building, individually housed in wire meshed cages (dimensions: 30 × 20 × 35 cm) under a 12 : 12 h light-dark cycle until marketing and ambient temperature (21 to 25°C). All rabbits were kept under the same management, hygienic, and environmental conditions and had access to water *ad libitum*. During the whole experimental period, the feed intake was determined precisely. Feed samples were taken to analyse for proximate components using the methods of [7] for determining moisture, crude protein (CP), crude fiber (CF), ether extract (EE), and ash. Neutral detergent fiber procedures were performed according to Van Soest, et al., [8].

### 2.2. Sample Collection and Preparation

Blood samples were collected via anterior vena cava puncture from 5 animals selected randomly in each treatment. The samples were centrifuged at 3,000 g and 4°C for 15 min to separate out the serum and were stored at −20°C until analysis. Other blood samples were collected in EDTA tubes from the same animals for packed cell volume, total and differential white blood cell counts, red blood cell counts, and hemoglobin concentration. The samples were determined using standard laboratory procedures. At the end of trials, fresh luminal digests from five rabbits per group were chosen randomly and subjected to a stream of CO_2_ in bottles and transferred immediately to the laboratory and stored at −80°C until the gut microbial composition analysis. The microbial counts (total bacterial count, *Clostridium* spp, and *Enterococcus*) were determined according to the methods of [9].*E. coli* were identified by the methods described by [10]. The serotyping of *E. coli*isolates was done by methods described [11]. The serum antioxidant-related indices, including the total antioxidant capacity (T-AOC), superoxide dismutase (SOD), glutathione peroxidase (GSH-PX), catalase (CAT), and the concentration of malondialdehyde (MDA), were determined with commercially available reagent kits (Elabscience Biotechnology Inc., USA). Moreover, the levels of immunoglobulin (IgM, IgG, and IgA) in serum were detected by an enzyme-linked immunosorbent assay (ELISA) kit (Elabscience Biotechnology Inc., USA). All measurements were calculated at least in triplicate and according to the manufacturer's instructions. The haematology parameters were carried out according to the procedure of Drew, et al., [12]. Total protein, albumin, glucose, ALT, and AST were measured calorimetrically using commercial kits (ERBA, diagnostics Mannheim GmbH, Germany) according to the manufacturer's instructions.

### 2.3. *In vitro* Gas Production Technique and Sampling Procedure

The *in vitro* gas production (GP) technique was conducted to test bacterial activities in the digestive tract of rabbits with added *Ziziphus spina-christi* in their diets.

Fresh content of the gastrointestinal tract was obtained from 10 rabbits slaughtered from the control group, subjected to a stream of CO_2_, and then discharged immediately with special solutions under anaerobic conditions.

Total gas production was measured by incubating tested diet with the content of the digestive tract *in vitro*, following the methods of [13].

The sample of testing diets (weight 0.2 mg) of each was incubated for 48 hours in a bottle which had a size of 100 ml after measuring GP (ml gas). Cumulative gas production was measured at 3, 6, 12, 24, 36, and 48 hr. Ammonia (NH_3_-N) concentration was estimated by the method of [14]. The digesta (the solid part) were treated with the pepsin activity, according to [15] to estimate protein degradability.

### 2.4. Digestibility Trials

At the end of the experiment, digestibility trials were carried out to determine the nutrient digestibility and nutritive values. Rabbits were taken randomly (5 within each treatment). Animals were housed individually in cages (dimensions: 30 × 20 × 35 cm) that allowed the separation of feces and urine. All rabbits were kept under the same management, hygienic, and environmental conditions. The experimental diets were offered twice daily at 9 a.m. and 15 p.m., and fresh-water was provided *ad libitum*. The survey of daily feed consumption was recorded. Any possible feed contamination was removed from the feces. Samples of daily feces of each rabbit were taken and oven-dried at 60º C for 48 h and then were ground and stored for proximate chemical analysis. Samples of feces were analyzed for DM, CP, EE, CF, and ash, according to the classical A.O.A.C (2012) methods.

### 2.5. Statistical Analysis

Data were statistically analyzed using One-Way ANOVA with the means comparisons procedure [16]. Differences among means were tested by Duncan's multiple range test [17]. The following statistical model was used:

(1) $$\begin{array}{c} Yij=µ+ai+eij, \end{array}$$

where *Y* is the dependent variable; μ is the overall mean of observations; a is the effect; and *e* is the residual error.

## 3. Results and Discussion

Chemical compositions of testing diets are shown in Table 1. The organic matter content of ZL was higher. Chemical compositions are comparable in all of the content of the tested diet. The content of NDF into ZL was at a suitable level for rabbits. These data were in agreement with [18].

**Figure 1:**
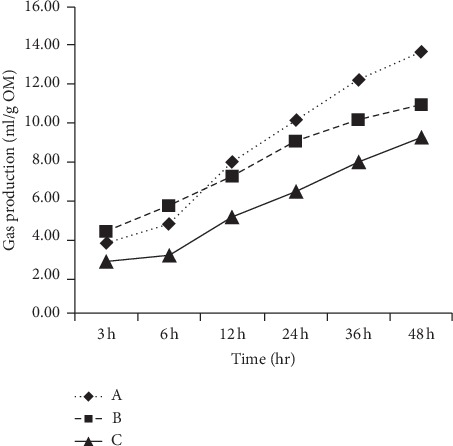
Volume (ml/g Organic matter) of cumulative gas produced when predigested diets were incubated with intestinal contents at different incubation times, where A = ZL0, B = ZL10), and C = ZL20); ZL0: control group, ZL10: 10 g ZL/kg, and ZL20: 20 g·ZL/kg.

Table 2 demonstrates the effect of ZL supplement on ammonia-N and crude protein degradability. There was a statistically significant difference in the ammonia-N between the three groups. Although the amount of gas production increased over time, the addition of ZL resulted in a decrease in the amount of gas produced compared to the control diet. Data illustrated in Figure 1 show the increase in gas production over time, quantities of gas released from the control diet were higher, and then the released gases decreased significantly (*P* < 0.05) with the addition of ZL. Incorporation of ZL in rabbit diets caused a decrease of GP in the digestive tract due to lower fermentation of microorganisms, and this observation is in agreement with the findings of [19]. These results are comparable, as a decrease in total bacteria count was found in both studies, and it indicates that adding ZL in rabbit diets has a positive effect on reducing gastrointestinal fermentation. Furthermore, the highest gas production rate was obtained in the control group (0.057 ml/h) and the lowest in the higher supplement of ZL (0.026 h^−1^). Due to the high content of ZL of condensed tannins (CTs), production of gases was affected because they inhibit the activity of bacteria in the digestive channel, and there are multiple hypotheses explain this effect. One hypothesis is that CT acts as a hydrogen sink [20]. Another hypothesis is that CT acts directly upon methanogens in the gastroenteritis [19]. A third hypothesis is that indirect inhibition occurs by decreasing the availability of nutrients to microorganisms; subsequently, substrate digestibility reduces, which indirectly inhibits microbial populations. The same trend was observed for the values of NH_3_-N.

The addition of a high level of ZL to rabbit diets led to a decrease (*P* < 0.05) in the total count of bacteria as well as the number of *E. coli* and *Clostridium* spp. These results may be attributed to that fact that tannins have general antimicrobial activities and have been reported to prevent the development of bacteria by precipitating microbial protein [19]. Korji [21, 22] has found that the aqueous extract of *Ziziphus spina-christi* leaves has shown significant antibacterial activity against *E. coli,* in comparison with eight antibiotics. The number of *Enterococcus* bacteria was not affected by supplementation as shown in Table 3. This can be attributed to the ability of CT in binding to minerals (Lavin [23], organic molecules such as proteins [24], carbohydrates [25], or lipids [26]. It is possible that CT bound to microbial enzymes modulate their activity [27].

Results in Table 4 demonstrate the haematological and serum biochemical indices of rabbits fed on a diet containing ZL. Haemoglobin parameters, white blood cell count and red blood cell count, of the control group and the groups ZL10 and ZL20 were compared. The same trend of the result was observed in WBC differentiation. The values of biochemical parameters obtained in this study were in the normal range of values defined for these parameters by previous studies [28] in clinical healthy rabbits. These observations of total protein and albumin within the range of reference values reported in healthy rabbits in previous studies [29] which reported that normal systemic protein utilization of the liver with feed of *Moringa* leaves. While glucose significantly decreased with the addition of ZL, AST in the blood increased significantly. These results are in agreement with the findings of [30] who indicated that blood glucose levels are not affected after supplementation of *Moringa oleifera* aqueous leaf extract in rats. The values of TP, albumin, and ALT measurements showed no significant differences among the groups that fed on test diets. It is likely possible that ZL has a positive effect on the health status of the rabbits. All values of kidney activities were within the normal ranges established [31].

The effects of ZL on the serum immunoglobulins are shown in Table 5. Compared with the control group, the high levels of ZL supplement lead to a significant increase (*P* < 0.05) in the serum IgA, IgG, and IgM level. Similar to these results, [32] found high immunity in the experimental animals with a feeding diet containing tannin-rich leaves.

Table 6 demonstrates the effects of supplementation with ZL on the activities of antioxidant enzymes in the serum of growing rabbits. Antioxidants expressed as T-AOC, GSH-Px, T-SOD, and CAT in the blood of animals fed on diets containing high levels of ZL were significantly higher. Higher serum T-AOC, T-SOD, and CAT activities were observed in rabbits supplemented with a high level of ZL compared with the control group (*P* < 0.05). The supplementation of ZL increased serum GSH-Px activity.

The addition of ZL to rabbit diets led to an increase in dry matter intake. The data on dry matter feed intake are presented in Table 7. These results could be attributed to the increase of the digestion of all nutrients. On the other hand, there was no significant change in the apparent digestion coefficient of DM, OM, CP, and fat. This is in agreement with the results of El-Sheikh, et al. [33], as they have found that feeding different levels of (0, 10, 20, and 40%) *Ziziphus spina-christi* leaf meal-containing diets did not affect DM, OM, CP, and EE digestibility.

## 4. Conclusions

Under the condition of the present study, the results suggest that dried ZL supplementation up to 20 g/Kg diet might improve the bacterial community, antioxidants, biochemical parameters and blood constituents of rabbits, and digestibility. Using dried ZLs should be considered when using other industrial materials are not feasible. Future studies are needed to confirm the potential benefits of ZLs found in this study.

## Figures and Tables

**Table 1:** Chemical composition of *Ziziphus spina-christi* leaves and differently tested diet (% on DM basis).

Item	ZSCL	ZL0	ZL10	ZL20
DM	92.62	93.76	93.30	93.47
OM	91.37	91.06	90.92	91.00
CP	8.50	16.94	16.84	16.90
EE	3.29	2.23	2.25	2.24
Ash	8.63	8.94	9.08	9.00
NDF	21.25	36.63	36.63	36.63

Berseem hay (35%), barley (32%), wheat bran (15%), rice bran (3%), soya bean meal (10%), molasses (4%), limestone (0.75%), common salt (0.15%), mineral and vitamin mixture, and anticoccidial (0.1%).

**Table 2:** Effect of ZL supplement on ammonia-N and crude protein degradability of the testing diet incubated in digestive tract content.

Item	ZL0	ZL10	ZL20	*P* value	±SE
Ammonia-N (g/kg OM)	2.17^a^	1.93^ab^	1.86^b^	0.05	0.073
Crude protein degradability (%)	54.20	52.72	52.08	0.74	1.950

^a, b, c^The same row having different superscripts differ significantly (*P* < 0.05). ZL0: control group, ZL10: 10g ZL/kg, and ZL20: 20 g·ZL/kg.

**Table 3:** Effects of dietary ZL supplementation on some ileal microbiota of growing rabbits.

Item	ZL0	ZL10	ZL20	*P* value	±SE
Total bacterial count (×10^6^)^1^	12.26^a^	11.80^b^	11.58^b^	0.02	0.125
*Escherichia coli* (×10^4^)^1^	6.13^a^	5.90^ab^	5.55^b^	0.04	0.121
*Clostridium* spp. (log CFU/g)	5.21^a^	4.72^b^	4.18^c^	0.001	0.106
*Enterococcus* (log CFU/g)	6.23	6.16	6.04	0.47	0.101

^a, b, c^The same row having different superscripts differ significantly (*P* < 0.05). ZL0: control group, ZL10: 10 g·ZL/kg, and ZL20: 20 g ZL/kg.

**Table 4:** Haematological and serum biochemical indices of rabbits fed on a diet containing ZL.

Item	ZL0	ZL10	ZL20	*P* value	±SE
*Blood cells*					
Haemoglobin (g/dl^−1^)	11.42	11.45	11.46	0.92	0.077
RBC (10^5^/1)	5.22	5.44	5.49	0.30	0.121
White blood cell (×10^9^/l)	9.16	9.35	9.44	0.40	0.136
Lymphocytes (%)	62.03	62.17	62.36	0.45	0.175
Neutrophils (%)	30.55	30.46	30.39	0.94	0.338
Monocytes (%)	3.07	3.07	3.08	0.98	0.074
Basophils (%)	2.63	2.63	2.64	0.98	0.063
Eosinophils (%)	1.72	1.65	1.52	0.95	0.466

*Blood chemistry*					
TP (g/dl^−1^)	6.14	6.26	6.32	0.16	0.060
ALB (g/dl^−1^)	2.62	2.67	2.70	0.83	0.093
Glucose (mg/dl)	71.25^a^	70.54^b^	70.21^b^	0.002	0.119
ALT (U/L)	23.00	23.12	23.34	0.38	0.165
AST (U/L)	14.72^b^	15.01^a^	15.16^a^	0.002	0.05

^a, b, c^The same row having different superscripts differ significantly (*P* < 0.05). ZL0: control group, ZL10: 10 g·ZL/kg, and ZL20: 20 g·ZL/kg.

**Table 5:** Effects of dietary ZL supplementation on the immune response of growing rabbits.

Item	ZL0	ZL10	ZL20	*P* value	±SE
Immunoglobulin A (IgA)	1.48^b^	1.54^b^	1.64^a^	0.007	0.024
Immunoglobulin G (IgG)	6.28^b^	6.90^b^	7.09^a^	0.002	0.099
Immunoglobulin M (IgM)	0.62^c^	0.71^b^	0.82^a^	0.0001	0.009

^a, b, c^The same row having different superscripts differ significantly (*P* < 0.05). ZL0: control group, ZL10: 10 g·ZL/kg, and ZL20: 20 g·ZL/kg.

**Table 6:** Effects of dietary ZL supplementation on antioxidants in the blood of growing rabbits.

Item	ZL0	ZL10	ZL20	*P* value	±SE
T-AOC	7.55^b^	8.01^ab^	8.37^a^	0.09	0.214
GSH-Px	479.89^b^	527.88^a^	531.94^a^	0.001	6.071
T-SOD	111.67^b^	122.83^a^	124.91^a^	<0.0001	0.721
CAT	5.94^c^	6.53^b^	7.32^a^	0.003	0.167

T-AOC total antioxidant capability, GSH-Px glutathione peroxidase GSH-Px, T-SOD total superoxide dismutase, CAT catalase. ^a, b, c^The same row having different superscripts differ significantly (*P* < 0.05). ZL0: control group, ZL10: 10 g·ZL/kg, and ZL20: 20 g·ZL/kg.

**Table 7:** Effects of dietary ZL supplementation on estimating apparent nutrient digestibility of growing rabbit diets.

Item	ZL0	ZL10	ZL20	*P* value	±SE
Dry matter intake (g/h/d)	1027.33^b^	1130.06^a^	1138.75^a^	0.001	13.332
Dry matter digestibility (%)	70.16	71.68	72.23	0.55	1.338
Organic matter digestibility (%)	72.20	73.74	74.31	0.44	1.136
Crud protein digestibility (%)	68.19	69.51	69.71	0.49	0.933
Fat digestibility (%)	76.88	78.34	78.95	0.29	0.869

^a, b, c^The same row having different superscripts differ significantly (*P* < 0.05). ZL0: control group, ZL10: 10 g·ZL/kg, and ZL20: 20 g·ZL/kg.

## Data Availability

The data used to support the findings of this study are available from the corresponding author upon request.
